# Deployment Design of Wireless Sensor Network for Simple Multi-Point Surveillance of a Moving Target

**DOI:** 10.3390/s90503563

**Published:** 2009-05-13

**Authors:** Kazuya Tsukamoto, Hirofumi Ueda, Hitomi Tamura, Kenji Kawahara, Yuji Oie

**Affiliations:** 1 Department of Computer Science and Electronics, Kyushu Institute of Technology, 680-4, Kawazu, Iizuka, 820-8502 Japan; E-Mails: kawahara@cse.kyutech.ac.jp; oie@cse.kyutech.ac.jp; 2 System Platforms Research Laboratories, NEC Corporation, 1753, Shimo-Numabe, Nakahara, Kawasaki, 211-8666 Japan; E-Mail: h-ueda@cb.jp.nec.com; 3 Network Design Research Center, Kyushu Institute of Technology, 680-4, Kawazu, Iizuka, 820-8502 Japan; E-Mail: tamu@ndrc.kyutech.ac.jp

**Keywords:** sensor network design, multi-point surveillance, moving target tracking, tracking probability, power consumption, sensor density

## Abstract

In this paper, we focus on the problem of tracking a moving target in a wireless sensor network (WSN), in which the capability of each sensor is relatively limited, to construct large-scale WSNs at a reasonable cost. We first propose two simple multi-point surveillance schemes for a moving target in a WSN and demonstrate that one of the schemes can achieve high tracking probability with low power consumption. In addition, we examine the relationship between tracking probability and sensor density through simulations, and then derive an approximate expression representing the relationship. As the results, we present guidelines for sensor density, tracking probability, and the number of monitoring sensors that satisfy a variety of application demands.

## 1. Introduction

The recent development of low-cost sensors [1, 2, 3] that are equipped with an embedded processor, sensing module, and radio transceiver, has led to the concept of wireless sensor networks (WSNs) in a ubiquitous network. Since these sensors communicate with each other by exchanging data with neighboring sensors, a WSN functions as an autonomous organization. WSNs have considerable potential for applications in a variety of areas, including business, environmental surveying [4] such as disasters, and military areas. One particular application that has received considerable attention in recent studies is the tracking of a mobile target, for example: video surveillance of humans and vehicles in urban areas [5].

However, there are a number of issues that need to be addressed for moving target tracking in WSNs. First, sensor nodes are subject to severe resource constraints such as low processing speed, small memory size, limited battery power, and a narrow and unreliable communication bandwidth. Most existing schemes for target tracking assume that the capability of individual sensors is very high, such that a sensor can determine the distances and/or directions of nearby sensors (and, thus, to a target) [6, 7]. However, the resource constraints on sensors necessitate a simple target-tracking algorithm. Second, as reported elsewhere [8], all sensors in a certain area are managed in a centralized manner, which means that a coordination node controls all of the sensors in that area. Due to these inherent constraints of WSNs, the existing approaches cannot provide scalability. Therefore, an algorithm operating in distributed manner is needed to enable an individual sensor to decide its own behavior based on localized and limited information. In addition, for continuous tracking of a high-speed moving target, monitoring sensor(s) need to switch smoothly along the path of the moving target. Thus, in the present paper, we focus on a simple distributed and continuous target-tracking algorithm for WSNs.

In a WSN consisting of a number of low-performance sensors, the information obtained from one sensor node may be of little or no use by itself. Therefore, by combining the data from neighboring sensors, also referred to as sensor fusion, more target information can be obtained, and high-resolution tracking can be achieved [9]. While some existing schemes monitor targets by using all nearby sensors, these sensors suffer high power consumption. Although several monitoring schemes have been proposed [10, 11], they focus on achieving either high accuracy or low power consumption. In reality, the appropriate resolution varies according to the application and user requirements. Given the considerations of monitoring resolution and power consumption, an algorithm to determine the appropriate number of sensors is necessary. This cannot be done in any of the existing schemes.

In the present paper, we discuss two simple and flexible target-tracking schemes for a distributed WSN: a primitive and a sophisticated schemes. We propose the use of the sophisticated scheme. Unlike those in previous studies, this scheme can select the appropriate number of monitoring sensors in a distributed manner with consideration of monitoring resolution and power consumption, and then monitor a target using these multiple low-performance sensors (multi-point surveillance). Moreover, the proposed scheme changes the monitoring sensors in response to the movement of the target. Through simulation experiments, we demonstrate that the proposed scheme can achieve (1) high tracking probability, (2) low power consumption regardless of the target's speed, and (3) flexibility with respect to the change in the number of required monitoring sensors.

Furthermore, when a WSN is actually constructed, a wide range of configurations, such as the state transition period in each sensor and the sensor density, should be evaluated for both the required tracking probability and power consumption, which differ according to the application. To the best of our knowledge, no existing studies mention a procedure to construct the WSN for multi-point surveillance of a moving target. Therefore, we examine the relationship between the tracking probability achieved by the proposed scheme and the sensor density. Then, through simulation experiments, we show the impact of the number of monitoring sensors on the performance. From this relationship, we derive an approximate expression for easy estimation of tracking probability. Finally, through the approximation, we demonstrate the deployment design of a WSN for multi-point surveillance of a moving target.

This paper is organized as follows. Section 2. describes related researches. Section 3. outlines our proposed multi-point surveillance scheme. In Section 4., we examine the performance of our proposed scheme in terms of tracking probability and energy consumption. Section 5. discusses the deployment design of a WSN based on the effects of both sensor density and the number of monitoring sensors. Concluding remarks are presented in Section 6.

## 2. Related research

Research on WSNs for target tracking has recently attracted considerable attention. Some studies, such as [6] and [12], have examined the network topology to reduce the communication cost to a sink point, which is a type of server in a WSN. However, more research efforts have focused on collaborative detection and tracking of the target. Although some studies have investigated signal processing, namely, location estimation of the target, most existing studies in this category have focused on the construction of an effective tracking structure using sensors in the WSN. These studies are mainly classified into the following six schemes: (1) naive [13], (2) periodic monitoring [14, 15], (3) single-sensor monitoring [16], (4) dynamic clustering [17, 18, 19], (5) prediction-based [20, 21, 22], and (6) distributed coordination [23, 24, 25, 26]. Each of these is discussed below.

**Naive:** All sensor nodes remain in the active state at all times and search/monitor the target in their sensing areas. The sensor nodes send data to the sink point periodically. As a result, the energy consumption in terms of each node and in terms of the entire WSN is high [13].
**Periodic Monitoring:** All sensor nodes change their states between the active and sleep states periodically and independently. When the sensor nodes are in the active state, they search for or monitor the target and report the sensing data. This scheme can reduce the total time in the active state of all of the sensors, but only when all of the sensor nodes and the base station are well synchronized. However, the high number of monitoring sensors is necessary, thus increasing energy consumption [14, 15].
**Single-sensor Monitoring:** Only one node that can detect the target becomes active. Whenever a node wakes up, the node continuously monitors the target until the target moves out of its sensing area. Consequently, all except one sensor node enter the sleep state to save energy. However, since only one node monitors the target, the required quality of information of the target cannot be satisfied. The tracking probability becomes low when the target moves at high speed. Moreover, the power consumption among the sensors becomes unbalanced, shortening the network lifetime [16].
**Dynamic Clustering:** The WSN consists of powerful nodes and low-end sensor nodes. One of the powerful nodes that receives the strongest signal from the target becomes the cluster head node and constructs a cluster using nearby sensor nodes. All nodes including the cluster head node perform continuous monitoring to improve the tracking probability. However, the tracking probability decreases where the nodes are sparsely deployed, because only the powerful nodes can be the cluster head. Furthermore, since rotation of the cluster head is not provided, the energy consumption of the cluster head is high, and power consumption between low-end sensor nodes is also unbalanced [17, 18, 19].
**Prediction-based:** The WSN consists of powerful nodes and low-end sensor nodes, like Dynamic Clustering scheme. Each powerful node predicts the next location of the target, and then wakes up an adjacent powerful node before the target leaves its own sensing area and enters the sensing area of the adjacent node by using a wake-up mechanism. If this scheme misses the target, it searches for the target throughout the entire WSN. Because this scheme wakes sensors based on the prediction results, the missing rate would be high and power consumption among sensors would also be unbalanced when the target changes its route beyond the prediction, e.g., random movement [20, 21, 22].
**Distributed Coordination:** The WSN consists of end sensor nodes only, that is, there is no powerful node such as a base station or cluster head in the WSN. These sensors dynamically establish group of nodes in a distributed manner and then track a moving object by using the coordination of multiple sensor nodes to reduce the energy consumption and improve the robustness of network connection. However, since the tracking of a moving target is extremely difficult due to the distributed management of their sensors, the tracking probability would be decreased [23, 24, 25, 26].

As stated in Section 1., (1) **distributed** (2) **multi-point surveillance** with (3) **simple sensors** is essential for obtaining detailed information of a moving target. Table 1 shows the architecture and sensor capability in existing target tracking schemes. The first two schemes (Naive and Periodic Monitoring) use simple sensor nodes with unintelligent behavior (no management) for general WSN applications. That is, although these schemes satisfy (2) and (3), they cannot achieve good performance in terms of tracking probability and power consumption due to its unintelligent behavior.

In contrast, the next three schemes (Single-sensor Monitoring, Dynamic Clustering, and Prediction-based) can achieve good tracking probability based on the assumption that all of the sensors in the WSN are managed in a centralized manner (rather than in a distributed manner), meaning that a coordination node, such as a base station or cluster head, controls all of the sensors in its area. However, it is difficult to deploy these powerful nodes in the WSN and to control them in a centralized manner. Moreover, these existing schemes generally assume that the capability of individual sensors is very high, for example: a sensor can detect the location of the target and can issue directions (wake up/shut down) to nearby sensors based on the prediction of the target's movement.

Based on these results, we believe that these centralized schemes do not provide scalability to the WSN. Thus, in the present paper, we focus on a distributed type of scheme in which an individual simple sensor decides its own behavior based on localized and limited information, thereby achieving multi-point surveillance with the appropriate number of monitoring sensors. Recently, several schemes providing the distributed coordination among multiple sensors have been proposed to track a moving target. In [23], a distributed event localization, tracking, and classification framework (DELTA) is studied.

In the DELTA, an event is detected or tracked by dynamically established groups. The group is managed by group leader, which is dynamically decided in a distributed manner and behaves as the dynamic cluster head. Ref. [24] proposed other distributed target tracking scheme by exploiting the pure peer-to-peer system, and therefore such a WSN is easy to build due to the simplicity of sensor deployment. However, the schemes proposed in [23] and [24] require the sensor nodes to know their location through various location services such as GPS and received signal strength, that is, high-performance sensor node is assumed, and thus their deployment cost will be high. On the other hand, Refs. [25] and [26] assume the limited capability of sensors, e.g., binary sensing for detecting the target, and determine the monitoring sensors in the distributed manner. In these schemes, although the angle information between neighboring sensors is required for deciding the monitoring sensors, they have not discussed how to get the location information at all.

Through these considerations, to the best of our knowledge, none of the existing schemes satisfies all the requirements. That is, no existing scheme selects the appropriate number of monitoring sensors with low-performance in a distributed manner while achieving good performance in terms of tracking probability (monitoring resolution) and power consumption.

## 3. Proposed multi-point surveillance scheme

As described in Section 1., multi-point surveillance with quick switching of monitoring sensors is essential for obtaining detailed information and achieving continuous monitoring of a moving target. Here, we propose two simple and flexible multi-point surveillance schemes with exactly *M* monitoring sensors, specified by the users or applications to detect moving targets in WSNs.

### 3.1. Capability and state transition of the sensor

**Figure 1.**
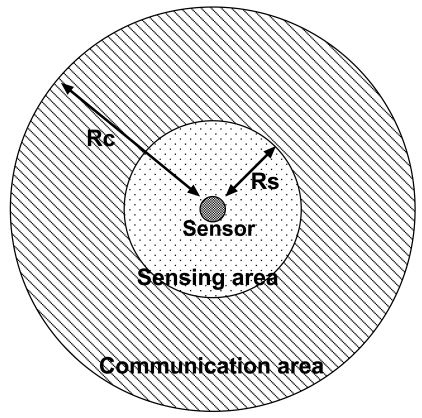
Sensor model.

A sensor node typically has two functions: *sensing* and *communication*. These capabilities are limited by the sensor's cost. Therefore, we assume that each sensor has only the most rudimentary capabilities of binary sensing and broadcast communication. Figure 1 shows a model of the sensor assumed in the present paper.

#### Binary sensing

A sensor can sense whether or not the target exists in its sensing area of radius *R_s_*.

#### Broadcast communication

The sensor can broadcast messages in the communication area of radius *R_c_* (=2*R_s_*).

**Figure 2.**
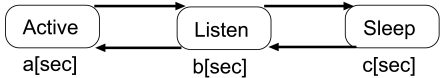
State transition model.

Today's sensor nodes are equipped with a state transition function for power savings. In the present paper, to achieve uniform power savings among all sensors, we assume that each sensor can autonomously and individually change its status at time intervals of *a, b, c* [*sec*] to one of the following three states, as shown in Figure 2: *Active, Listen*, and *Sleep*. Sensors in the *Active* state, which are referred to as *Active* sensors, can sense the target and can communicate with nearby sensors. Sensors in the *Listen* state (*Listen* sensors) can receive any messages sent from nearby sensors, but cannot send any messages. Sensors in the *Sleep* state (*Sleep* sensors) can neither sense nor communicate any messages.

### 3.2. Proposed scheme

In this section, we compare our two schemes: primitive and sophisticated. In our proposed schemes, since the low-performance sensors are assumed with considering the actual sensor products, we design the simplest algorithm for deciding the monitoring sensors by reducing the load of message exchange as much as possible. More specifically, we firstly assume that the sensors exchange messages with each other in a broadcast manner, thus they don't require the neighboring sensors' address information. Next, to reduce the complexity and the energy consumption of the proposed schemes, only two types of messages, i.e., ALERT and DETECT, are defined. To obtain detailed information of a moving target by low-performance sensors, the proposed sophisticated scheme reliably and quickly decides which multiple monitoring sensors to use by user demand and/or applications based on the ALERT and DETECT message exchanges.

#### Primitive scheme

The primitive scheme focuses only on the selection of monitoring sensors; that is, the movement of the target is not considered. The primitive scheme consists of two phases: **Detection** and **Selection**.

**Detection phase:** First, if an *Active* sensor detects a target in its sensing area, it immediately broadcasts the detection in an ALERT message to nearby sensors within communication range. Note that *Active* sensors broadcast the ALERT message whether or not the target has already been detected by other sensors. Specifically, an *Active* sensor that initially could not detect the approach of the target during the *Active* state sends the ALERT message when it finally detects the target.
**Selection phase:** After the detection phase, all *Active* and *Listen* sensors that received the ALERT message set their received message counter to 1, immediately change their status to the *Active* state, and begin sensing. Sensors that cannot detect the target return to the *Listen* state almost simultaneously. The *Active* sensors decide the delay time randomly from 0 to 1 [sec] to avoid collisions with frames sent by other *Active* sensors. If the random delay timer expires, the *Active* sensor first compares the number of ALERT and DETECT messages of *M* sensors, received during the random delay time. If the number of received messages is smaller than *M*, that is, *M* monitoring sensors are not yet defined, the sensor broadcasts a DETECT message and remains in the *Active* state. In contrast, if the number of received messages exceeds *M*, the sensor changes its state to *Listen*.

In this way, the primitive scheme selects *M* monitoring sensors by utilizing the ALERT/DETECT messages. Each monitoring sensor changes its state to *Listen* when the target cannot be detected as a result of the target movement. However, since this primitive scheme does not consider the movement of the target in detail, it has the following three serious problems in the case of a moving target.

1. **State synchronization:** All of the sensors that can receive an ALERT message but cannot detect the target change to the Listen state almost simultaneously, i.e., synchronization occurs. At this time, there are no Active sensors near the target except for M active sensors. Therefore, when the target moves away from the area that the M active sensors are monitoring, no other sensors can detect the target. Thus, it may be difficult to track the target continuously.
2. **Large power consumption and delay:** In the primitive scheme, all sensors that receive an ALERT message change to the Active state. As a result, the number of state transitions increases drastically. Typically, state transition requires some power consumption. Therefore, the lifetime of the WSN can be shortened and its performance can be degraded.
3. **Collision:** In this scheme, the waiting delay is a purely random value between 0 and 1 [sec], and so collision avoidance is not guaranteed, even if the value is large. If a collision occurs, M sensors cannot be selected exactly, thereby decreasing the tracking probability of the M sensors and increasing the power consumption.

#### Sophisticated scheme

**Figure 3.**
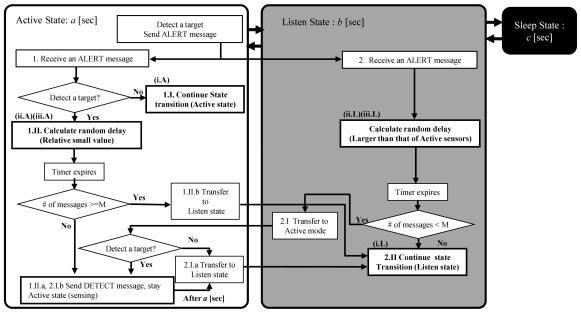
Monitoring sensor decision flow.

We enhance the primitive multi-point surveillance scheme to address these problems. By using the flow diagram shown in Figure 3, we describe how the monitoring sensors are decided after an Active sensor broadcasts an ALERT message; at this point, the detection phase is the same as that in the primitive scheme. However, we modify the behavior of the *Active* and *Listen* sensors during the **selection phase**.

1. Each *Active* sensor that receives the ALERT message decides its status based on whether it detects the target.
  1.I. **Not detected:** It remains in the *Active* state for some duration after expiration of the predetermined period, since the approach of the target can be inferred from the received messages.
  1.II. **Detected:** It calculates the slotted random delay time (less than *ActMax* × slot time) and decides its behavior in response to the number of messages received when the delay time has expired. Note: slot time and *ActMax* are described later.
    1.II.(a) **Less than** *M*: The *Active* sensor broadcasts a DETECT message and continues to monitor the target.
    1.II.(b) **More than** *M*: The *Active* sensor changes to the *Listen* state without broadcasting a DETECT message.
2. Each *Listen* sensor that receives the ALERT message immediately calculates the random delay time, unlike the *Active* sensors. Note that we set the random time within range of ([ActMax-LisMax] × slot time). *LisMax* is described later. As a result, *Listen* sensors decide their status after the decision of the *Active* sensors. More specifically, the *Listen* sensors decide their status in response to the number of messages received at the expiration of the delay time.
  2.I. **Less than** *M*: The *Listen* sensor detects the lack of monitoring sensors, and immediately changes to the *Active* state. Then, the activated sensors from the *Listen* state decide their own status based on whether they detect the target.
    2.I.(a) **Not detected:** The activated sensor returns to the *Listen* state.
    2.I.(b) **Detected:** The activated sensor broadcasts a DETECT message and starts target monitoring.
  2.II. **More than** *M*: The *Listen* sensor remains in the *Listen* state and follows the state transition cycle, as shown in Figure 2. In such cases, they do not need to change to the *Active* state, which means that the additional power consumption associated with frequent state transitions can be avoided.

In this manner, the sophisticated scheme attempts to keep an individual sensor's state transition sequence unchanged (Figure 3 (1.I, 2.II)), thereby avoiding state synchronization. However, in Figure 3 (1.I), we add 2 [sec] to the rest period of the *Active* state to detect the target leaving the area of the *M Active* sensors. The choice of an appropriate additional time is beyond the scope of the present paper and will be considered in future research.

Finally, we modify the random delay time, that is, the function of message transmission control. For access to a wireless channel, some types of MAC layer protocols, such as CSMA/CA of IEEE 802.15.4 and CSMA of TinyOS, have been employed in recent sensor products. Moreover, some enhanced protocols have been developed. Therefore, the proposed scheme should be designed to work well with any MAC protocol. Thus, to avoid collisions with other messages, each of the sensors calculates the random delay based on the slot time with consideration of the transmission time.

The random delay time is calculated as follows:

(1) Random delay time=Random value×Slot time,

(2) $$\text{Slot time}=\frac{\text{Data size}}{\text{Data rate}}+\text{Propagation Delay}.$$

In equation (1), we set a different range of random values between the *Active* sensors and the *Listen* sensors. The maximum value for the *Active* sensors is referred to as *Act max*, and that for the *Listen* sensors is referred to as *Lis max*.

## 4. Tracking probability and energy consumption

We evaluated the proposed multi-point surveillance schemes in terms of tracking probability and power consumption to examine the effect of the moving speed of the target.

### 4.1. Simulation model

**Figure 4.**
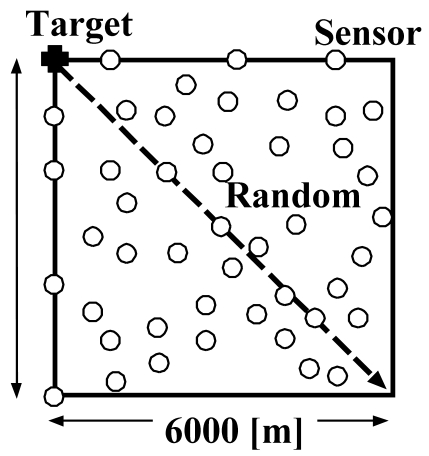
Simulation model.

The simulation parameters are listed in Table 2. We assume that the sensor network is deployed in a 6, 000 [m] × 6, 000 [m] square area and that one-million sensors are randomly deployed in this area, as shown in Figure 4. Initially, equal numbers of *Active/Listen/Sleep* sensors are deployed. In this area, a target moves in a straight line at a constant speed from the upper left corner to the lower right corner. The WSN then monitors and tracks the moving target with exactly *M* sensors. In the simulation, we first assume *M* to be 3, that is, one moving target can be monitored by three active sensors. We then vary M from 2 to 5.

Here, we consider the maximum number of monitoring sensors that are necessary to detect and track the target with exactly *M* sensors in the simulation environment. The total number of sensors that can detect the target, *X*, and the average number of *Active* and *Listen* sensors that can detect the target after receiving an ALERT message, *Y,* are calculated as follows by using the variables listed in Table 2:

(3) $$X=\pi R_{s}^{2}\times \left(\frac{N}{6,000^{2}}\right),$$

(4) $$Y=\frac{a+2b}{a+2b+c}\times X.$$

Therefore, to track the target with *M* sensors, *Y* should at least satisfy the following equation:

(5) Y≥M.

In our simulation, since we assumed that the sojourn times of each state are the same (Table 2), *Y* is $\frac{2}{3}\times X$. When we set *N* to 1, 000, 000, *X* is approximately 8.5, and *Y* is 5.6, based on Eqs. (3) and (4). From equation (5), the maximum number of monitoring sensors (*M*) is 5. Therefore, the value of *M* is varied from 2 to 5 in our simulation (Because *Y* is 5.6 described before, M = 2 means that (*Active/Listen*) sensor density is sufficient (dense). In contrast, M = 5 means that density is at the lower limit (sparse)).

In the present paper, we investigate the effectiveness of the proposed scheme for tracking probability and power consumption. The tracking probability is examined by two performance measures: average tracking probability *P_t_* and handover index *I_h_*. In addition, the state power consumption *E_s_* and the power consumption for communication *E_c_* are employed as performance measures. Note that we do not consider the power consumption for state transition due to a lack of information. These performance measures are calculated as follows:

(6) $$P_{t}=\frac{\text{Sum of tracking period with}\;M\;\text{sensors}}{\text{Simulation time}},$$

(7) $$I_{h}=\frac{\text{Avg}.\text{of sensing duration}\;(M\;\text{sensors})}{\text{Average interval of ALERT messages}},$$

(8) $$E_{s}=\sum_{i\in \{\text{active},\text{listen}\}}e_{i}\times T_{i},$$

(9) $$E_{c}=\frac{\sum_{j\in \{tx,rx\}}e_{j}\times C_{j}}{P_{t}}.$$

The ratio of the interval time of ALERT messages to the monitoring time of the same *M* active sensors, i.e., *I_h_*, directly affects the tracking probability. If the ALERT message is transmitted after the target moves from the area in which *M* active sensors are monitoring, *I_h_* becomes less than 1, and so the tracking probability decreases. That is, *I_h_* is a performance measure of the ability to detect a target. The term *E_s_* is the sum of the state power consumption of all of the *Active* and *Listen* sensors receiving ALERT messages during the selection phase in Section 3.2., and *e_i_* is the state power consumption for 1 second. *T_i_* is the total sojourn time throughout the simulation time, and *E_c_* is the average power consumption for the message exchange (transmit (tx) and receive (rx)) of one sensor, which is necessary for improving the 1% tracking probability. Finally, *e_j_* is the power consumption for transmitting (tx)/receiving (rx) a message, and *C_j_* is the number of transmitting/receiving messages. In our simulation, since we assume that the message length is set based on ZigBee specification [27], short message of 10 bytes including address, frame type, and message sequence number is employed.

### 4.2. Simulation results

In this section, we evaluate the performance of the proposed schemes (primitive scheme and sophisticated scheme) through simulation experiments. In the present paper, as we would like to investigate the fundamental performance of the proposed scheme, that is, realistic wireless effects such as fading, shadowing, and interference from other devises are not considered at all, we develop a new simple simulator from the scratch to achieve this. Our main concern is enabling the proposed schemes to provide high tracking probability with low power consumption, even when the target moves at high speed. First, we assume *M* is 3. Then, we investigate the impact of *M* (sensor density) on the flexibility of the proposed schemes; that is, *M* is varied from 2 to 5.

#### Tracking probability

**Figure 5.**
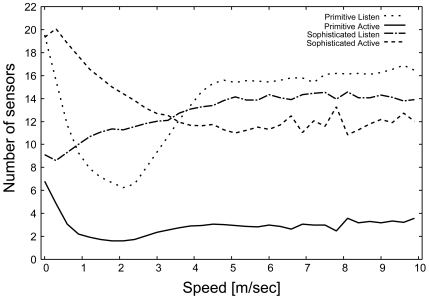
Number of *Active* and *Listen* sensors.

Figure 5 shows the average number of *Active* and *Listen* sensors immediately after selecting the monitoring sensors (*M*) in the primitive and sophisticated schemes. In the primitive scheme, the number of *Listen* sensors becomes significantly larger than the number of *Active* sensors. The state synchronization problem described earlier causes an imbalance between the number of *Active* and the number of *Listen* sensors. In contrast, in the sophisticated scheme, the number of *Active* and *Listen* sensors becomes stable and similar with the increase in the speed of the target. This result indicates that the sophisticated scheme can solve the state synchronization problem even when the target moves at high speed.

**Figure 6.**
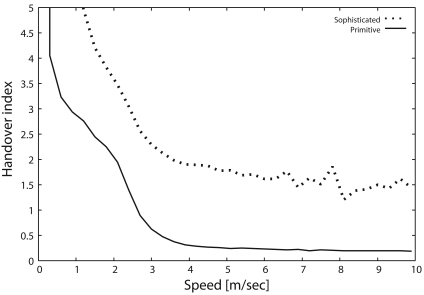
Handover index, *I_h_*.

Next, we show the handover index (*I_h_*) as a function of the target speed in Figure 6. Figure 6 indicates that *I_h_* with the primitive scheme decreases drastically with the increase in target speed and then becomes less than 1 when the target speed exceeds 2.5 [m/s] due to the state synchronization problem (Figure 5). In contrast, the sophisticated scheme can keep the *I_h_* above 1 even when the target moves at high speed, because the synchronization problem is completely solved by the sophisticated scheme. Hence, the sophisticated scheme can achieve an *I_h_* of 1.5, which is six times larger than that of the primitive scheme whenever the target speed exceeds 4 [m/s] (up to 10 [m/s]).

**Figure 7.**
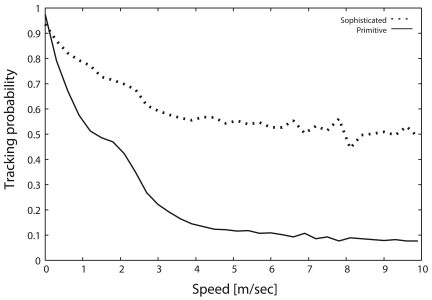
Tracking probability, *P_t_*.

Figure 7 shows the tracking probability as a function of the target speed. In this figure, the tracking probability with the primitive scheme drastically degrades as the target velocity increases. As shown in Figures 6 and 7, the tracking probability with the primitive scheme decreases in response to the decrease of *I_h_*. In contrast, the sophisticated scheme can maintain a relatively high tracking probability even when the target moves at high speed. In particular, the tracking probability of the sophisticated scheme is greater than 50 %, which is six times larger than that of primitive scheme, whenever the target speed exceeds 4 [m/s] (up to 10 [m/s]). Based on these results, the sophisticated scheme drastically improves the handover index by introducing message transmission control, described in Section 3.2., thereby drastically improving the tracking probability.

#### Energy Consumption

Next, we compare the energy consumption in the primitive and sophisticated schemes. In the present paper, we classify the energy consumption into two parts (state and message exchange) and calculate *E_s_* and *E_c_* using Eqs. (8) and (9), respectively. The values of *e_i_* and *e_j_* employed here are listed in Table 3. These values are calculated from Refs. [2] and [3], respectively. Based on these considerations, we examine the effectiveness of the proposed schemes in terms of energy consumption, as listed below.

##### State energy

**Figure 8.**
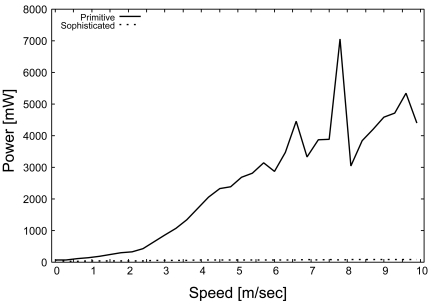
Power consumption (state), *E_s_*.

Figure 8 shows the effect of the moving speed of the target on *E_s_* in the primitive and sophisticated schemes. Figure 8 indicates that the power consumption of the primitive scheme increases drastically when the target moves at high speed. In the primitive scheme, each sensor waits for a random delay time from 0 to 1 [sec] to avoid collisions with frames sent by other *Active* sensors. Therefore, the latency until the required number of sensors for monitoring are selected (*E_t_*) increases, and thus *E_s_* of the primitive scheme becomes significantly larger than that of the sophisticated scheme. Furthermore, the number of ALERT messages increases in response to the increase in target speed. As a result, *E_s_* increases drastically.

In the sophisticated scheme, Figure 8 shows that *E_s_* becomes extremely low, independently of the change in target speed. The random delay time in the sophisticated scheme is calculated based on the slot time relative to the transmission time. Hence, the latency until selecting the monitoring sensors (*E_t_*) becomes significantly shorter than that of the primitive scheme. As a result, the sophisticated scheme significantly reduces the state power consumption.

##### Communication energy

**Figure 9.**
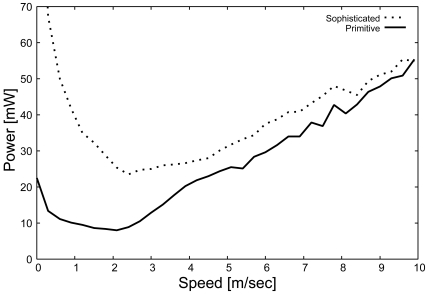
Power consumption (message exchange), *E_c_*.

Figure 9 shows the impact of the moving speed on the power consumption for message exchange in the primitive and sophisticated schemes. Figure 9 indicates that the power consumption of the primitive scheme becomes relatively low and gradually increases in response to the increase in target velocity. In the primitive scheme, since all of the sensors that can receive the ALERT message but cannot detect the target change to the *Listen* state almost simultaneously, successive transmission of ALERT messages rarely occurs. As a result, the power consumption becomes relatively low.

Figure 9 also shows that the power consumption of the sophisticated scheme is relatively large compared to that of the primitive scheme and gradually increases in a manner similar to the primitive scheme. In the sophisticated scheme, all sensors that can receive the ALERT message, but cannot detect the target, remain in the *Active* state for some time. These *Active* sensors promptly detect the approach of the target and transmit ALERT messages. As a result, the transmission and reception of messages caused by these ALERT messages consume more power than do the transmission and reception in the primitive scheme. However, when the target moves at high speed, the difference between the primitive and the sophisticated scheme becomes small. These results indicate that the sophisticated scheme can achieve good power consumption performance for a moving target. In particular, when the target moves at high speed, the sophisticated scheme achieves excellent power consumption performance for message exchange. Finally, the sophisticated scheme can achieve relatively high tracking probability with low power consumption under moderate density.

#### Impact of *M*

So far, we assumed the number of required monitoring sensors (*M*) to be set to a fixed value (*M*=3). Therefore, we here vary *M* from 2 to 5, and examine the flexibility of the sophisticated scheme with respect to the number of monitoring sensors. More specifically, because *Y* in equation (5) is 5.6, *M* = 2 indicates that the (*Active*/*Listen*) sensor density is sufficient (dense). On the other hand, *M* = 5 indicates that the density is at the lower limit (sparse).

**Figure 10.**
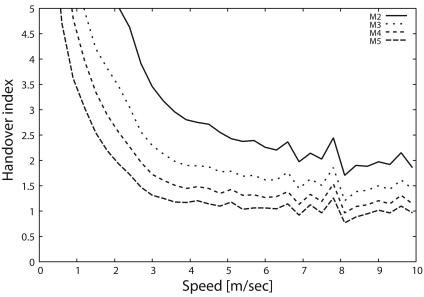
Impact of *M* on handover index (*I_h_*).

**Figure 11.**
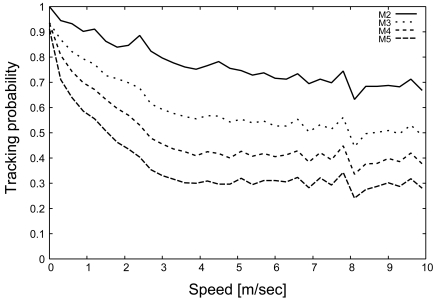
Impact of *M* on tracking probability (*P_t_*).

Figures 10 and 11 show the relationship of the change of *M* on the handover index and tracking probability, respectively. As shown in Figure 10, when *M* is set to be small, the handover index is large. Although the handover index is higher than 1 in most cases, it is lower in other cases, such as for a target speed of 10 [m/s] and *M* = 5. In this case, from Eq (5), the density of sensors is not sufficient for the required number of monitoring sensors. However, Figure 11 indicates that a tracking probability of 30% is achieved by the sophisticated scheme due to the state maintenance and message transmission control. Therefore, the sophisticated scheme can provide flexibility with respect to the density of sensor deployment.

Moreover, comparing Figures 10 and 11, we demonstrate that 50% of the tracking probability can be achieved when the handover index is higher than 1.5. In particular, the sophisticated scheme can achieve a tracking probability of 70% for *I_h_* = 2. The relationship between the handover index and the tracking probability is beyond the scope of the present paper and will be investigated in a future study.

## 5. Discussion of sensor deployment design

In the previous section, we examined the tracking probability and power consumption under our proposed schemes (primitive and sophisticated schemes). Through simulations, we showed that the sophisticated scheme, in response to the target movement, can achieve (1) high tracking probability, (2) low power consumption, and (3) quick decisions of the monitoring sensors. Although the achievable tracking probability depends dynamically on the sensor density, we have not examined the impact of the density. Therefore, in this section, we first examine the effects on the tracking probability due to (i) sensor density and (ii) number of monitoring sensors. Finally, we consider the deployment design of a WSN that satisfies the user requirements effectively. As shown later in the present paper, the sophisticated scheme becomes the proposed scheme.

### 5.1. Simulation model

This section describes the simulation model employed here to investigate the effects of the sensor density and number of monitoring sensors on the tracking probability by using our proposed scheme. The simulation model and parameters are almost the same as those shown in Figure 4 and Table 2, respectively. The differences in simulation parameters are listed in Table 4. The total number, *N_all_*, of sensors (*Active, Listen, Sleep*) are randomly deployed in the area shown in Figure 4. Initially, equal numbers of *Active*, *Listen*, and *Sleep* sensors are deployed. A target moves on the dotted line in Figure 4 at a fixed speed of 9.7 [m/sec]. Then, tracking probability *P_t_*, defined as the performance measure, is given by equation (6), presented above.

Obviously, the change in the state transition period of a sensor has a strong influence on the relationship between the sensor density and tracking probability. Hereinafter, we assume a fixed state transition period, and then set the period of *Active, Listen*, and *Sleep* to 4, 2, and 4 [sec], respectively, so that each state has the same duration time on average.

### 5.2. Simulation results and deployment design

Through simulation experiments, we first examine the relationship between the tracking probability and the sensor density when the number of monitoring sensors (*M*) is given by a user or an application. From this, we derive an approximate expression of the tracking probability and the sensor density for estimating the sensor density that can satisfy any tracking probability of the user or application. Next, we examine the effect of the number of monitoring sensors (*M*), thereby extending the approximate expression to estimate the required sensor density under any number of monitoring sensors and tracking probabilities. Finally, we clarify the deployment design of a WSN.

#### Relation between sensor density and tracking probability

Here, the minimum sensor density that can satisfy the required tracking probability is examined in detail. Here, we target both the number of *Active* and *Listen* sensors, i.e., *N_AL_*, and then examine the approximate relationship between *N_AL_* in 100 *m*^2^, i.e., density of *N_AL_*, and the tracking probability. Note that *Sleep* sensors that do not have an impact on the performance are not included in the sensor density.

##### Simulation results

**Figure 12.**
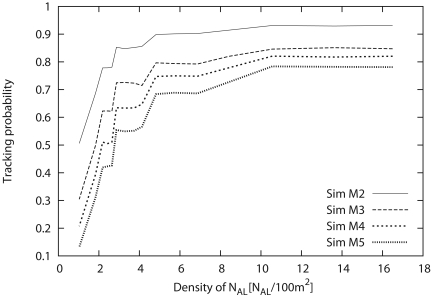
Effects of sensor density on tracking probability.

Figure 12 shows the tracking probability as a function of the sensor density when the number of monitoring sensors (*M*) is varied from 2 to 5. From this figure, we can see that the tracking probability drastically increases in response to the increase of sensor density, and the tracking probability approaches some value beyond some sensor density. As a result, we can confirm that an excess increase of sensor density does not improve the tracking probability. When the sensor density is relatively small, the number of sensors that can detect the target tends to be less than *M*. However, the sensors can detect the movement of the target easily with the increase of sensor density, thereby drastically increasing the tracking probability. Conversely, we can see that the maximum tracking probability decreases with the increase of *M*.

Next, we consider the sensor density that can achieve the maximum tracking probability. Although the sensor density that can achieve convergent tracking probability in Figure 12 satisfies the user/application requirement, the sensor density should be as small as possible from the viewpoint of cost. Therefore, we focus on the sensor density that converges to some value, that is, the minimum sensor density. Through general estimation, we briefly conclude that it is necessary for the number of *N_AL_* to be at least six times that of the monitoring sensors (*M*). In this way, the required number of sensors that satisfies the requirement can be estimated through Figure 12; that is, the necessary sensor density can be derived according to the user/application requirement.

##### Estimation of approximate formula

Next, we use nonlinear regression to derive the approximate numerical formula that represents the relationship shown in this section. We apply equation (10) as the regression formula (where *x* is the density of *N_AL_*), and thus the regression factors are analyzed from the simulation results (Figure 12). Table 5 shows the factors of equation (10) derived by the approximation.

(10) $$P_{t}=A\times (1-\text{exp}(-B\times x)).$$

**Figure 13.**
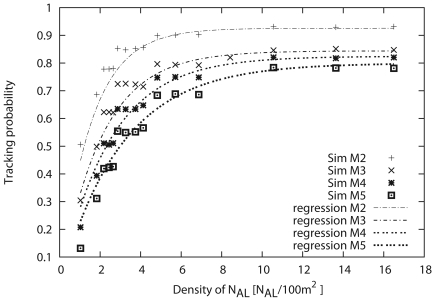
Comparison between simulations and approximate formula representing the relationship between sensor density and tracking probability.

From Table 5, we can see that factors of both *A* and *B* decrease with the increase of the number of monitoring sensors (*M*). Figure 13 compares the simulation results with the approximate formula. In Figure 13, the tracking probabilities calculated from the formula are almost the same as the simulation results. That is, the performance under our proposed scheme can be estimated by this formula. From these results, we confirm that the necessary number of sensors can be estimated by using this formula when the number of monitoring sensors and the tracking probability are given. Consequently, we can reliably build WSNs that satisfy the user and application requirements.

#### Impact of the number of monitoring sensors, *M*

So far, we examined the relationship between the sensor density and the tracking probability when the number of monitoring sensors *M* is varied from 2 ∼ 5. Although *M* is variable, the required sensor density needs to be estimated based on both the (a) tracking probability and (b) number of monitoring sensors. Therefore, we examine the relationship between them, then derive the approximate numerical formula for this relationship. The simulation parameters of the sensor density (*N_all_* and *N_AL_*) employed here are listed in Table 6.

##### Simulation results

**Figure 14.**
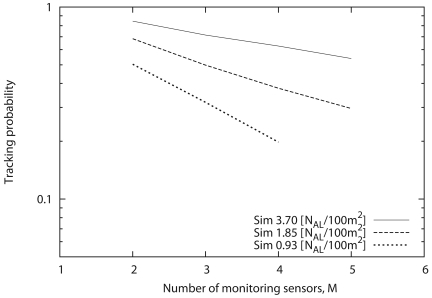
Effect of *M* on tracking probability.

Figure 14 shows the relationship between the number of monitoring sensors and the tracking probability. As shown in this figure, the tracking probability exponentially decreases with the increase in the number of monitoring sensors. The increase in *M* causes a drastic decrease in the common sensing area among the *M* sensors. As a result, we suppose the following formula represents the relationship between them:

11 $$P_{t}=C\times D^{M}.$$

##### Estimation of approximate formula

Next, we derive the factors *C* and *D* in equation (11). These factors in Table 7 are obtained by applying the nonlinear regression from the simulation results in Figure 14. As can be seen in Table 7, when the sensor density doubles, factor *C* decreases by approximately 0.07, whereas factor *D* increases by approximately 0.11. From this, we can state that there is some correlation between these factors and the sensor density.

**Figure 15.**
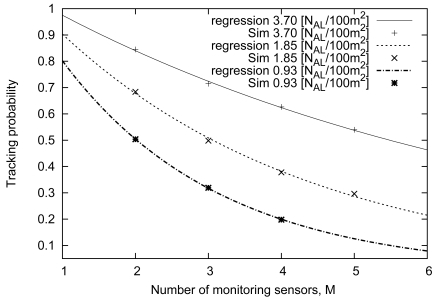
Comparison between the approximate formula and simulation results : Effect of the number of monitoring sensors.

Figure 15 compares the simulation results with the approximate formula. As shown in Figure 15, these values show a similar trend. As a result, we can derive a reliable approximate formula for the relationship between the number of monitoring sensors and the tracking probability.

#### Discussion of approximate formula and deployment design

Finally, we discuss the relationship of the tracking probability and both the number of monitoring sensors and the number of deployment sensors. More specifically, we integrate the approximate formulas derived thus far in order to estimate the required sensor density with consideration of both the tracking probability and the number of monitoring sensors. From this, we discuss the deployment design of WSNs for multi-point surveillance.

We first discuss the integration of the formulas based on equation (10). Factor *A* in equation (10) indicates the maximum tracking probability, and it varies with *M*. Therefore, when the relationship between the tracking probability and *M* in Figure 13 is applied as the relationship between *A* and the *M*, we derive the following formula:

(12) $$A=0.9514\times 0.9698^{M}.$$

Then, we discuss factor *B*. From the results obtained from a regression analysis, we suppose that factor *B* follows the progression of differences with *M*. Therefore, we use the following formula as the relationship between *B* and *M*,

(13) $$B=0.7838-\sum_{i=2}^{M}0.5026\times {\left(1/2\right)}^{i-2}.$$

From these results, we verify that the relationship between parameters, sensor density, *M*, and tracking probability, follows equation (14). In other words, we can estimate one parameter (sensor density, the number of monitoring sensors, and tracking probability) based on the other two parameters:

(14) $$\begin{array}{l} P_{t}=A\times (1-\text{exp}(-B\times x)),\\ A=0.9514\times 0.9698^{M},\\ B=0.7838-\sum_{i=2}^{M}0.5026\times {\left(1/2\right)}^{i-2}. \end{array}$$

In this way, the number of monitoring sensors *M* is the dominant effect on the sensor density at the maximum tracking probability, which exponentially decreases with the increase of *M*. In contrast, when *M* is large, we show that high sensor density is necessary to improve the tracking probability. Furthermore, the sensor density for achieving any number of monitoring sensors and tracking probability can be estimated by using equation (14).

## 6. Conclusions

In the present paper, we proposed a simple multi-point surveillance scheme for the moving target in WSNs. The proposed scheme can satisfy the following two requirements to achieve moving target tracking with high resolution: (I) multi-point surveillance with an appropriate number of sensors, and (II) dynamic and quick decision making of monitoring sensors in response to the movement of the target. In the present paper, we proposed two new multi-point surveillance schemes. Basically, a sensor that detects the target broadcasts the ALERT message, and other sensors that can receive this message and detect the target then send the DETECT message to monitor the target with the number of sensors required by the user.

We first proposed a simple distributed scheme, which we call the primitive scheme. However, three problems were found in the primitive scheme. Therefore, based on the primitive scheme, we proposed a second scheme, which we call the sophisticated scheme, to address these problems. Simulation results revealed that the sophisticated scheme can achieve high tracking probability with low power consumption in the state and message exchange even for a high-speed moving target under randomly deployed sensor networks, due to (a) message transmission control and (b) maintenance of the state transition cycle. Since the sophisticated scheme can provide flexibility with respect to the change in number of monitoring sensors, the sophisticated scheme proposed here is considered to be practical for general WSNs.

Then, we examined the relationships among (1) sensor density, (2) number of monitoring sensors, and (3) tracking probability under our proposed multi-point surveillance scheme (sophisticated scheme). Two approximate formulas that express the relationship of sensor density and number *M* of monitoring sensors with the tracking probability were derived from the simulation results. From these formulas, we can state that the tracking probability greatly increases with the increase of the sensor density. However, it tends to converge beyond some sensor density. In addition, the tracking probability exponentially decreases with the increase of the number *M*.

We integrated the two formulas to estimate the appropriate sensor density for achieving both tracking probability and the number of monitoring sensors. As a result, we showed the deployment design of WSNs that can achieve distributed multi-point surveillance for a moving target.

Currently, we are conducting a detailed examination of the relationship with tracking probability. More specifically, we are investigating the relationship in terms of sensor capability (sensing/ communication), state transition period of a sensor, and moving speed of a target, in addition to the sensor density and number of monitoring sensors *M* discussed in the present paper. In the future, we will propose a general expression including these relationships. Furthermore, practical implementation of the proposed scheme using a real sensor product (Mote sensor) is currently being carried out, and the results of implementation of the proposed scheme will be presented in the near future.

## Figures and Tables

**Table 1.** Architecture and sensor's capability employed in existing target tracking schemes.

	Management		Monitoring sensor		Sensor's capability	
	Centralized	**Distributed**	Single	**Multi**	**Low**	High
Naive	×	×	×	○	○	×
Periodic Monitoring	×	×	×	○	○	×
Single-sensor Monitoring	○	×	○	×	×	○
Dynamic Clustering	○	×	×	○	×	○
Prediction-based	○	×	×	○	×	○
Distributed Coordination	×	○	×	○	×	○ (Δ [25, 26])

**Table 2.** Simulation parameters.

Simulation time	600 [sec] (10 [min])
Simulation area	6,000 [m] × 6,000 [m] (square area)
Number of sensors in the WSN	*N* = 1,000,000
Required number of sensors	*M* = 2 - 5
Sensing Range	*R_s_* = 10 [m]
Communication Range	*R_c_* = 20 [m]
State Transition	*Active* (*a*):*Listen* (2*b*):*Sleep* (*c*) = 1:1:1 (*a* = 4 [sec], *b* = 2 [sec], *c* = 4 [sec])
Speed of target	0 [m/s] - 10 [m/s]
Message length	10 [bytes]
Time slot	400 [*μ*sec]
Slot range	Act_max = 30, Lis_max = 128
Bandwidth	250 [Kb/s]

**Table 3.** Power consumption.

State		Message exchange	
*Active*	30 mW/sec	Transmit	56.7 mW/message
*Listen*	21.6 mW/sec	Receive	62.91 mW/message

**Table 4.** Simulation parameters.

Density of *N_all_*	1.4 ∼ 25 [*N_all_/*100*m*^2^]
Density of *N_AL_*	0.93 ∼ 16.67 [*N_AL_/*100*m*^2^]
Speed of target	9.7 [m/sec]

**Table 5.** Factors calculated from nonlinear regression.

*M*	A	B	Decrease in B
2	0.9137	0.7838	–
3	0.8407	0.5359	0.2479
4	0.8391	0.3855	0.1504
5	0.8269	0.2984	0.0871

**Table 6.** Parameters of sensor density.

*N_all_*, Density of *N_all_*	[0.5/1/2]-million, [1.4, 2.8, 5.6]*/*100 m^2^
Density of *N_AL_*	[0.93, 1.85, 3.7]*/*100 m^2^

**Table 7.** Estimated factors by nonlinear regression.

Sensor density [#/100 *m*^2^]	*C*	*D*
0.93	1.2745	0.6290
1.85	1.2035	0.7504
3.70	1.1315	0.8616
